# Endosymbiotic theory of aging revisited: Age-related leakage of mitochondrial dsDNA/RNA stimulates cytosolic nucleic acid sensors which remodel the immune network and promote the aging process

**DOI:** 10.1007/s10522-026-10470-9

**Published:** 2026-07-07

**Authors:** Antero Salminen, Kai Kaarniranta, Anu Kauppinen

**Affiliations:** 1 Department of Neurology, Institute of Clinical Medicine, University of Eastern Finland, P.O. Box 1627, 70211 Kuopio, Finland; 2 Department of Ophthalmology, Institute of Clinical Medicine, University of Eastern Finland, P.O. Box 1627, 70211 Kuopio, Finland; 3 Department of Ophthalmology, Kuopio University Hospital, P.O. Box 100, 70029 KYS Kuopio, Finland; 4 School of Pharmacy, Faculty of Health Sciences, University of Eastern Finland, P.O. Box 1627, 70211 Kuopio, Finland

**Keywords:** Ageing, Bacterial origin, CGAS-STING, Endosymbiosis, Evolution, DNA/RNA sensors

## Abstract

About 1.5–2 billion years ago, an endosymbiosis between aerobic α-proteobacteria and anaerobic archaeal cells generated mitochondria, i.e., organelles capable of producing oxidative energy. The bacterial genome was fundamentally reduced and a circular mitochondrial genome evolved containing mainly the genes coding for the subunits of the electron transport chain. Before the symbiotic event, there existed a virus-host co-evolution which involved the development of sensors for detecting dangerous viral DNA/RNA molecules. Endosymbiosis supplied eukaryotic cells not only with an oxidative powerhouse to allow the evolution of more complex multicellular organisms but it also meant that cells now housed an organelle which was able to generate reactive oxygen species (ROS) and to leak mitochondrial DNA (mtDNA) and double-stranded RNA (dsRNA) into the cytoplasm. There is now abundant evidence that during aging and age-related diseases mitochondria are prone to release both mtDNA and dsRNA. In the cytoplasm, mtDNA/dsRNA molecules activate a number of cytosolic nucleic acid sensors leading to the secretion of type-1 interferons (IFN) and many other cytokines which promote an age-related proinflammatory state. Currently, it is known that mtDNA can activate the cGAS-STING pathway, AIM2 inflammasomes, IFI16 receptors, and ZBP1 sensors and in addition mitochondrial dsRNA stimulates RIG-1/MDA5 signaling. Interestingly, there is abundant evidence that all these receptors are drivers of cellular senescence and inflammaging. For decades, there has been mounting evidence that mitochondria have a crucial role in the aging process. We will examine this question from the perspective of evolution and propose that mitochondrial evolution created an endogenic source for the leakage of dangerous mtDNA/dsRNA which subsequently stimulated cytosolic DNA/RNA sensors, an evolutionarily conserved viral defence mechanism. It seems that these two evolutionary events provided not only the basis for the inevitable process of aging but also ensuring the death of parental organisms.

## Introduction

The endosymbiosis between aerobic α-proteobacteria and anaerobic archaeal cells was a crucial event in the evolution of eukaryotic cells, about 1.5–2 billion years ago (Sagan 1967; Fitzpatrick et al. 2006). The bacterial genome provided a potent mechanism for oxygen-dependent energy production which enabled the development of multicellular organisms. Eukaryotic cells contain two genomes, i.e., one is the nuclear genome while the other is the mitochondrial genome. After endosymbiosis, there occurred a major reduction and reorganization in the genome of α-proteobacteria to create the mitochondrial circular genome which contains only 37 genes encoding 13 polypeptides including essential subunits of the respiratory chain complexes (Taanman 1999). It is important to recall that many mitochondrial proteins, e.g., Krebs cycle enzymes, are encoded by the nuclear genome. Interestingly, there is convincing evidence that mitochondria have a crucial role in the aging process, i.e., it can be argued that they would be able to drive the aging process. There are several mitochondrial theories of aging proposing different mechanisms (Harman 1972; Miquel et al. 1980; Salminen et al. 2014a; Sun et al. 2016; Chen et al. 2020). The four most popular theories are (i) the mitochondrial free radical theory, (ii) mutations in the mitochondrial DNA (mtDNA), (iii) reduced mitophagy, and (iv) Krebs cycle intermediates induced landscape modification of the nuclear genome. However, there have appeared many conflicting results indicating that these mechanisms are not the primary cause of the aging process although they can promote the actual process and even trigger age-related diseases.

**Fig. 1:**
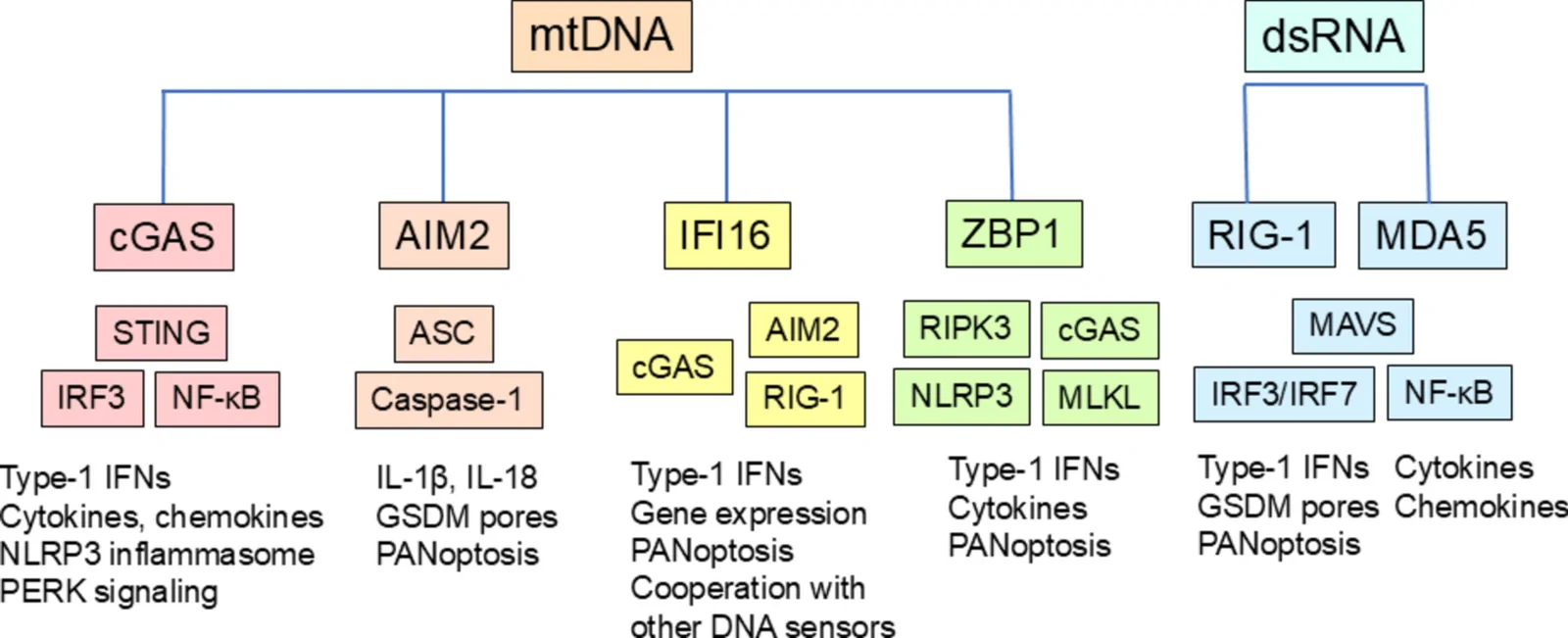
Leakage of mitochondrial mtDNA and dsRNA activates a number of nucleic acid receptors in the cytoplasm and stimulates several signaling mechanisms which enhance the age-related proinflammatory state and cell death via PANoptosis. *AIM2* absent in melanoma 2, *ASC* apoptosis-associated speck-like protein containing a CARD, *cGAS* cyclic GMP-AMP synthase, *dsRNA* double-stranded RNA, *GSDM* gasdermin, *IFI16* interferon-γ inducible protein 16, *IFN* interferon, *IRF* interferon regulatory factor, *MAVS* mitochondrial antiviral signaling protein, *MDA5* melanoma differentiation-associated protein 5, *MLKL* mixed lineage kinase domain like pseudokinase, *mtDNA* mitochondrial DNA, *NF-κB* nuclear factor-κB, *NLRP3* NLR family pyrin domain containing 3, PANoptosis, inflammatory cell death integrating pyroptosis, apoptosis, and necroptosis, *PERK* protein kinase R-like endoplasmic reticulum kinase, *RIG-1* retinoic acid-inducible gene-1, *RIPK3* receptor-interacting serine/threonine-protein kinase 3, *STING* stimulator of interferon genes, *ZBP1* Z-DNA-binding protein 1

Before the endosymbiotic event, there existed a virus-host coevolution which gradually evolved over a course of around 4 billion years ago (Koonin et al. 2022). The appearance of viral DNA/RNA molecules in the cytoplasm of archaeal cells represented a danger signal for cells and therefore there was a need to develop defence mechanisms against these pathogen-associated molecular patterns (PAMPs). There are several cytosolic nucleic acid receptors which act as sensors for these viral PAMPs and activate defence mechanisms (Zahid et al. 2020). Interestingly, these cytosolic sensors not only identify viral nucleic acids but they are also able to recognize mtDNA/dsRNA released from mitochondria. There is abundant evidence that with aging and cellular senescence, there is an increase in the leakage of mtDNA/dsRNA into the cytoplasm through different mitochondrial channels/pores (Panel et al. 2018; Zhong et al. 2022; Rottenberg 2023; Victorelli et al. 2025). In the cytoplasm, mtDNA/dsRNA molecules activate diverse nucleic acid sensors which stimulate sterile inflammatory responses by producing type-1 interferons (IFN) and several cytokines (Kim et al. 2023; Jung et al. 2025; Victorelli et al. 2025) (Fig. 1). First, we will clarify the endosymbiotic origin of mitochondria and then review many investigations indicating that the aging process and cellular senescence increase the leakage of mtDNA/dsRNA from mitochondria. Next, we will examine the properties of the most important mtDNA/dsRNA sensors and evaluate their role in the age-related activation of inflammatory responses. Finally, we will emphasize the relevance of the theory on endosymbiosis as a driver of the aging process.

## Endosymbiotic theory of aging

In 1967, Lynn Margulis (then Sagan) proposed the endosymbiotic theory on the evolution of eukaryotic cells suggesting that mitochondria were originally aerobic bacterial ancestors which were engulfed by anaerobic archaeal host cells (Sagan 1967). Her symbiosis view of eukaryotic cell evolution was disputed not only when it appeared but for many years afterwards, as discussed in many reviews (Poole and Penny 2007; Martin et al. 2015; Lazcano and Pereto 2017). The theory contained many thorny cellular and biological speculations, e.g., about the possibility of an endocytotic process as well as genetic questions related to the reduction of the bacterial genome compared to the mitochondrial genome. However, currently the endosymbiotic theory of mitochondrial origin is generally accepted (Gray 2012; Archibald 2015). Interestingly, the evolution of aging has aroused a number of theories which have been under active debate (Flatt and Partridge 2018; Li et al. 2023; Mc Auley 2024). Currently, there are two lines of experimental evidence highlighting the role of mitochondria as crucial agents in the aging process, i.e., mitochondrial dysfunctions have an important role in the proinflammatory phenotype of senescent cells (Wiley et al. 2016; Martini and Passos 2023; Hruby and Higuchi-Sanabria 2025) as well as mitochondria regulate many age-related responses of the innate immunity (Chowdhury et al. 2022; Marchi et al. 2023; Xu et al. 2025).

### Bacterial origin of mitochondria

Phylogenetic studies have revealed that mitochondria originated from the α-proteobacteria, probably from the group of Rickettsiales (Fitzpatrick et al. 2006; Fan et al. 2020). It has been estimated that a symbiotic event occurred about 1.5–2 billion years ago although adaptation continued for a long time. There are several properties which indicate that mitochondria have a bacterial origin, (i) mitochondria possess a small circular dsDNA without any histones similarly as in bacteria, (ii) mitochondria can divide independently of the host cell, (iii) mitochondrial ribosomes resemble those of bacteria, (iv) mitochondrial cristae display a similarity to the intracytoplasmic membranes present in α-proteobacteria, (v) the inner membrane of mitochondria resembles its counterpart in bacteria, whereas the outer membrane is reminiscent of the endocytic vesicles of host cells, and (vi) both mitochondria and bacteria are able to secrete vesicles (Munoz-Gomez et al. 2017; Roger et al. 2017; Zachar and Boza 2020; McMillan and Kuehn 2021; Picca et al. 2023a; Bravo-Arevalo 2025). The symbiosis conferred many benefits for anaerobic archaeal cells because aerobic bacteria allowed them a possibility to utilize oxygen for energy production. However, the transition of α-proteobacteria to mitochondria required a massive reduction of their genome size which was achieved by relocating many important mitochondrial genes to the host genome (Roger et al. 2017). The human mitochondrial genome, about 16.5 kb, is inherited exclusively from the mother and it contains the major genes for oxidative phosphorylation (Taanman 1999). Thus, mitochondria are fundamentally dependent on the imported proteins encoded in the host nucleus in order to maintain their structural and functional properties.

Although the size of circular mtDNA is small, mitochondria contain several copies of mtDNA both wild-type and mutated copies, i.e., this is called mitochondrial heteroplasmy (Stewart and Chinnery 2015; Aryaman et al. 2019; Parakatselaki and Ladoukakis 2021). The mutation rate of mtDNA is considerably higher than that of the nuclear genome which means that mutated copies can accumulate within the mitochondria, e.g., with aging and in many diseases (Sondheimer et al. 2011; Elorza and Soffia 2021; Green et al. 2025). Normally mitochondria contain two to ten copies of mtDNA although the number and size of mitochondria are highly variable and tissue-dependent. Rath et al. (2024) reported that the copy number of mtDNA significantly varied across human and mouse tissues, being highest in the heart and skeletal muscles. They observed about a 50-fold variation between human tissues and over 200-fold interindividual differences. There are several investigations indicating that the copy number of mtDNA in tissues declines significantly in many pathological states (Castellani et al. 2020). It is evident that the regulation of mitochondrial integrity by mitophagy has a major role in the regulation of both mtDNA copy number and heteroplasmy.

### Mitochondrial theory of aging

Given that mitochondria are the powerhouse of eukaryotic cells, it seems evident that mitochondria will play an important role in the aging process. Mitochondrial energy metabolism decreases with aging which indicates that the efficiency of mitochondrial function is disturbed during the aging process. Harman (1972) proposed the now well-known mitochondrial free radical theory of aging (MFRTA) since mitochondria are the primary site of ROS generation. An age-related increase in the production of ROS disturbs not only the function of mitochondria but it increases oxidative stress in tissues, a common hallmark of the aging process (Maldonado et al. 2023). Miquel et al. (1980) proposed that an increase in the generation of ROS induced mtDNA mutations which are potent drivers of age-related decline in the function of mitochondria. However, Trifunovic et al. (2005) generated a mutator mouse which displayed a robust increase in mutations in its mtDNA and caused an aging phenotype without any increase in the generation of ROS. Moreover, investigations of long-lived naked mole rats, a rodent species living over 30 years, revealed a high level of oxidative damage in their tissues indicating that oxidative stress did not prevent their longevity (Andziak et al. 2006). There has been critical debate about whether mutations in mtDNA are able to stimulate the aging process, e.g., the defects in mutator mice seem to destabilize the maintenance of nuclear genome and thus promote the aging process (Hämäläinen et al. 2019). Nonetheless, it does seem that the theory of the vicious cycle in mitochondria between ROS generation and mtDNA mutations does not explain the aging process.

Many other hypotheses have been presented related to the impaired function of mitochondria. For instance, Krebs cycle enzymes, located in the matrix of mitochondria, generate many intermediates, such as citrate, 2-oxoglutarate, itaconate, and succinate, which are not only potent immunoregulators but they can also modify the epigenetic landscape in the nuclear genome (Kaelin and McKnight 2013; Salminen et al. 2014a, b; Williams and O’Neill 2018; Borkum 2023). It is known that the aging process is associated with robust changes in the epigenetic landscape which affects the aging process and longevity (Han and Brunet 2012; Salminen et al. 2014a; Kane and Sinclair 2019). Currently, the role of mitochondria in this process is unknown, because (i) the enzymes of Krebs cycle are encoded in nuclei and (ii) the age-related changes in the cooperation between mitochondria and nuclei needs to be clarified. This topic is outside the scope of this review. Mitochondria have many other properties which can directly affect the aging process. For instance, mitochondria, similarly to α-proteobacteria, secrete extracellular vesicles (EV) which also contain mtDNA (Lazo et al. 2021; McMillan and Kuehn 2021; Picca et al. 2023a). However, Lazo et al. (2021) demonstrated that the amount of mtDNA in plasma EVs was significantly decreased with aging which indicates that either aged mitochondria are not prone to secrete EVs or mtDNA is released into cytoplasm during the aging process. It is known that the content of mtDNA in mitochondria declines with aging in human blood and skeletal muscle (Short et al. 2005; Zhang et al. 2017). In summary, there is robust evidence that a leakage of mtDNA from mitochondria is significantly increased with aging and this can stimulate inflammatory responses and promote the aging process.

### Age-related mtDNA/dsRNA leakage from mitochondria

The aging process is associated with robust changes in the structural and functional properties of mitochondria. There are several hallmarks of the mitochondrial population in aging tissues (i) clear morphological changes, such as fragmentation of mitochondria due to increased fission, (ii) a progressive decline in energy production, (iii) increased numbers of deletions and mutations present in mtDNA, (iv) an increased generation of ROS, (v) a decline in mitophagy and impaired mitochondrial quality maintenance, (vi) an increase in the permeability of mitochondrial membranes, and (vii) an elevated release of mtDNA/dsRNA (Seo et al. 2010; Amigo et al. 2016; Sun et al. 2016; Chen et al. 2020; Rottenberg 2023; Xu et al. 2025). Moreover, there are known to be different age-related changes which occur in the mitochondrial populations between various cell types and tissues. Currently, there is also a debate about whether mitochondrial dysfunctions trigger cellular senescence or vice versa. In fact, a mild mitochondrial stress stimulates mitohormesis which surprisingly can extend the organismal lifespan (Cheng et al. 2023; Gunawan et al. 2025). A slight mitochondrial stress in *C. elegans*, e.g., ROS exposure, stimulates the mitochondrial unfolded protein response (mtUPR) which can induce a chromatin reorganization in nuclei and promote longevity (Tian et al. 2016; Shpilka and Haynes 2018). Currently, it is known that in mammalian cells mitochondrial stress can stimulate both the mtUPR and an integrated stress response (ISR) which act in cooperation to increase mitochondrial ability to combat stress, i.e., enhance mitohormesis (Cheng et al. 2023; Gunawan et al. 2025). In mitohormesis, mitochondrial Lon protease P1 (LonP1) has a crucial role in the maintenance of mitochondrial proteomic integrity as well as it is a multifunctional guardian of mitochondrial homeostasis (Pinti et al. 2015; Zanini et al. 2023). LonP1 is a proteolytic enzyme which not only cleans the mitochondrial matrix from damaged proteins but it can also maintain mtDNA nucleoids and thus prevent mtDNA leakage. In *C. elegans*, the knockout of LonP1 increased oxidative stress, disturbed the maintenance of mitohormesis, and significantly reduced lifespan (Taouktsi et al. 2022). Interestingly, there is clear evidence that the expression of LonP1 declines in cellular senescence and with aging in different tissues (Bota et al. 2002; Bakala et al. 2003; Koltai et al. 2012; Zhang et al. 2024a). This confirms many investigations indicating that the maintenance of mitochondrial homeostasis declines with organismal aging.

An increase in the permeability of the mitochondrial inner membrane is a characteristic age-related alteration found in mitochondria (Rottenberg and Hoek 2021; Panel et al. 2018; Rottenberg 2023). This has been attributed to an opening of the mitochondrial permeability transition pores (mPTP) which allow the influx of water and small-molecular compounds into the mitochondria matrix. The influx of water and electrolytes can lead to swelling of mitochondria and induce a rupture of the outer mitochondrial membrane (OMM) (Sesso et al. 2012). An age-related increase in the mitochondrial generation of ROS and a calcium overload have key roles in the opening of mPTP in the inner mitochondrial membrane (IMM) (Bernardi et al. 2023; Giordano et al. 2025; Zhang et al. 2025b). Currently, the exact mechanism for the release of mtDNA from the mitochondrial matrix into the cytoplasm still needs to be clarified. However, it is known that the opening of mPTP in the IMM is associated first with the oligomerization and then the opening of the voltage-dependent anion channel (VDAC) in the OMM. The VDAC oligomers are mitochondrial gatekeepers by binding to many proteins and mtDNA and forming a larger mPTP/VDAC pore (Xian et al. 2022; Giordano et al. 2025; Zhang et al. 2025b). For instance, Xian et al. (2022) demonstrated in mouse macrophages that oxidized mtDNA was exported via the mPTP/VDAC-dependent channels. There are also two other types of mitochondrial pores, i.e., the BAK/BAX megapores and the gasdermin (GSDM)-induced pores, which can transport mtDNA/dsRNA into the cytoplasm (Cosentino et al. 2022; Krieger et al. 2024; Giordano et al. 2025). It is known that the BAX and BAK proteins, enhancers of apoptosis, can form megapores into the OMM across which mtDNA can leak into the cytoplasm and subsequently stimulate the dsDNA sensors (Cosentino et al. 2022). GSDMs are pore-forming proteins which are activated by caspase-1 to form pores into the OMMs and IMMs and thus triggering a leakage of mtDNA and subsequently leading to pyroptotic cell death (Miao et al. 2023; Zhu et al. 2024). There is abundant evidence that the level of cytoplasmic mtDNA increases with aging in both immune and non-immune cells (Zhong et al. 2022; Newman and Shadel 2023; Jimenez-Loygorri et al. 2024). The amount of circulating mtDNA also increases with aging, probably due to an increase in the numbers of dying cells (Pinti et al. 2014). It is not only mtDNA but mitochondrial dsRNA can also leak from mitochondria. Lopez-Polo et al. (2024) demonstrated that different human senescent cells released mitochondrial dsRNA into the cytoplasm thus stimulating their proinflammatory phenotype.

In addition to the mitochondrial channels, the clustering of mtDNA with nucleoid proteins affects the release of mtDNA into the cytoplasm. In the mitochondrial matrix, mtDNA is packed into compact protein-DNA complexes referred to as nucleoids (Garrido et al. 2003; Lee and Han 2017). Human mitochondrial nucleoids contain several nucleoid-associated proteins (NAP), such as mitochondrial transcription factor A (TFAM), mtDNA helicase (TWINKLE), DNA polymerase (POLG), LonP1 protease, and high mobility group proteins (HMG) (Garrido et al. 2003; Gilkerson et al. 2013; Lee and Han 2017). The TFAM protein has a key role in the organization and function of nucleoids because when it binds to a single copy of mtDNA, it becomes involved in its replication, transcription, and maintenance (Campbell et al. 2012; Kukat et al. 2015). There are investigations which have indicated that the binding activity of TFAM to mtDNA robustly declines with aging in mitochondria of rat brain and liver (Picca et al. 2013; Chimienti et al. 2019). Recently, Ding et al. (2025) demonstrated that a deficiency of TFAM induced the leakage of mtDNA into the cytoplasm in mouse intestinal ischemia–reperfusion injury. These studies indicate that the binding of mtDNA to the TFAM protein has a crucial role in its leakage from mitochondria.

The amount of mtDNA/dsRNA leakage from mitochondria is dependent on the quality control of mitochondria and nucleoids (Busch et al. 2014; Liu et al. 2025). Mitophagy is a selective mechanism to remove dysfunctional mitochondria and thus prevent a leakage of mtDNA (Ashrafi and Schwarz 2013; Onishi et al. 2021; Narendra and Youle 2024). There are two basic mechanisms which recognize damaged mitochondria, i.e., the ubiquitin-mediated PINK/Parkin pathway and the receptor-driven BNIP3/NIX axis. Several studies have revealed that inhibition of mitophagy in different experimental models promotes a leakage of mtDNA into the cytoplasm where it activates dsDNA sensors (Zhong et al. 2022; Jimenez-Loygorri et al. 2024; Qin et al. 2024; Zhou et al. 2025). Brunk and Terman (2002) were the first investigators to present the mitochondrial-lysosomal theory of aging which highlighted the role of imperfect autophagocytosis. Subsequently, abundant evidence has accumulated indicating that a robust decline in mitophagy significantly promotes the aging process and age-related diseases (Chen et al. 2020; Picca et al. 2023b). Currently, mitophagy is an important target in drug discovery research and for instance, there are several promising drug candidates acting through this mechanism being tested against neurodegenerative diseases (Antico et al. 2025). Interestingly, during the recent years, several specific mechanisms have been discovered which can eliminate mitochondrial nucleoids and damaged mtDNA via selective autophagy (Sen et al. 2022; Liu et al. 2024). Liu et al. (2024) demonstrated that the TFAM protein was able to act as an autophagy receptor which helped to eliminate any leaked mtDNA via an autolysosomal degradation. The TFAM protein contains the LC3 interacting region (LIR) by which the TFAM/mtDNA complex binds to the LC3B protein in the cytoplasm and subsequently it is degraded by selective autophagy. This pathway is called nucleoid-phagy and it prevents the activation of dsDNA sensors in the cytoplasm. Sen et al. (2022) reported that the H2O2-induced mtDNA damage in muscle cells triggered a remodeling of mitochondrial membrane and consequently transported endosomes to a close proximity of the nucleoid sub-compartment in mitochondria. The mitochondrial SAMM50 protein stimulated BAK clustering and the transfer of the nucleoid/mtDNA complex through the BAK channel into early endosomes where it was subsequently degraded in late autophagy vesicles. These examples indicate that mitophagy is a major mechanism to remove dysfunctional mitochondria, although in specific stress situations mutated mtDNA can be targeted to selective autophagy. With aging the activity of mitophagy declines and mtDNA can be released into the cytoplasm where it stimulates dsDNA sensors and promotes inflammatory responses.

## Leakage of mtDNA/dsRNA activates cytosolic nucleic acid sensors

Viruses are an important source of life-threatening danger for mammalian species and thus there exist a large number of pattern-recognition receptors (PRR), both for DNA viruses and RNA viruses. Recently, Jung et al. (2025) reported that there is a total of twenty-six different receptors which can be clustered into different groups although there is a degree of redundancy. The major difference is that distinct receptors can only recognize either dsDNA or dsRNA structures. Moreover, the receptors can be categorized according to their location in cells or cell types, their pattern recognition mechanisms, and finally which signaling pathways they activate. However, the cytosolic dsDNA and dsRNA sensors stimulate very similar antiviral immune responses including type-1 interferons (IFN) and proinflammatory cytokines. Zahid et al. (2020) and Jung et al. (2025) have classified cytosolic nucleic acid receptors into different groups and described their functional properties. Interestingly, as described above, the leakage of mtDNA and dsRNA from mitochondria can be recognized by cytosolic viral DNA and RNA sensors and subsequently they stimulate inflammatory responses. This means that mitochondrial disturbances are a potent source of inflammatory responses (Chowdhury et al. 2022; Marchi et al. 2023). Here, we will introduce those DNA/RNA sensors which are known to be involved in the regulation of cellular senescence and the aging process.

### cGAS-STING pahway

The cGAS-STING signaling pathway is an evolutionarily conserved defence mechanism in eukaryotic cells against infections and thus it has been intensively studied and recently reviewed in detail (Guo et al. 2025; Zhang et al. 2025a; Salminen et al. 2025a). The cGAS and STING proteins are normally ubiquitously expressed at a low-level and with low specificity in most non-immune cells, whereas the expression level is clearly higher in endothelial and immune cells (Human Protein Atlas). Briefly, cGAS-STING signaling involves different stages, (i) the recognition of mtDNA by the cGAS sensor in the cytoplasm, (ii) the activation of the cGAS enzyme catalyzes the formation of cyclic guanosine monophosphate (GMP)-adenosine monophosphate (AMP) dinucleotide (cGAMP) from GMP and AMP, (iii) the cGAMP dinucleotide is a messenger molecule which activates the STING protein in the endoplasmic reticulum, (iv) after transport to the Golgi complex the STING dimers act as a platform for the interaction of different coactivators which can trigger both the canonical and non-canonical pathways. The canonical pathway acts via the interferon regulatory factor 3 (IRF3) and nuclear factor-κB (NF-κB) signaling pathways generating type-1 IFNs as well as diverse cytokines and chemokines. The two non-canonical responses stimulated by STING signaling involve the activation of the NLR family pyrin domain 3 (NLRP3) inflammasomes and the protein kinase R-like endoplasmic reticulum kinase (PERK) signaling pathway (Wang et al. 2020; Zhang et al. 2022) (Fig. 1).

There is abundant evidence that mtDNA can stimulate the cGAS enzyme and subsequently trigger the STING signaling pathway which is able to remodel the immune network in different experimental conditions (Aarreberg et al. 2019; Huang et al. 2020; Willemsen et al. 2021; Hu and Shu 2023; Kim et al. 2023; Li et al. 2024). However, there are still many important unanswered questions about the function of mtDNA/cGAS-STING signaling, e.g., (i) while cGAS is a nuclear protein bound to the nucleosomes (Michalski et al. 2020; Pathare et al. 2020), its translocation into cytoplasm needs to be clarified, (ii) the non-canonical functions of the cGAS enzyme, and (iii) a clarification of the many activities of the STING protein and its translocation into nuclei (Dixon et al. 2021). Under physiological conditions, the cGAS protein is sequestrated into the chromatin structures where nucleosomes inhibit its activity (Michalski et al. 2020; Pathare et al. 2020). However, it is known that the cGAS protein is involved in DNA repair and cell cycle processes (Lu et al. 2024). A loss of nuclear integrity and chromatin disruption trigger the nuclear export of the cGAS protein into cytoplasm via the CRM1 nuclear export system (Sun et al. 2021). In cytoplasm, the cGAS protein is able to recognize mtDNA in a sequence-independent manner and catalyze the generation of cGAMP dinucleotides (Hall et al. 2017; Zhang et al. 2025a). Subsequently, the cGAMP molecule stimulates the STING signaling pathway. Interestingly, cGAMP can also act as an intercellular messenger and activate STING signaling in neighbouring cells (Xie and Patel 2023). This kind of function has been called paracrine communication, for instance, it occurs in an extension of cellular senescence within tissues (Admasu et al. 2021). The regulation of cGAS signaling is stringently regulated by post-translational modifications and certain coactivators, such as Z-DNA binding protein 1 (ZBP1) (Yu and Liu 2021; Lei et al. 2023; Zhang et al. 2025a). Interestingly, type-1 IFNs are able to stimulate the expression of the cGAS protein (Ma et al. 2015), i.e., there exists a positive feedback regulation between the cGAS-STING pathway and type-1 IFN signaling.

The STING oligomers organize a signaling platform in the Golgi complex where the STING proteins interact with different signaling proteins stimulating both immune and non-immune responses (Balka and De Nardo 2021; Zhang et al. 2025a). The canonical signaling involves two pathways, i.e., (i) the TBK1-driven IRF3 pathway and (ii) the IKKε-activated NF-κB signaling (Fig. 1). The IRF3 transcription factor stimulates the production of type-1 IFNs which promote the expression of IFN-stimulated genes (ISG) via the IFN receptor (IFNR) 1 and 2-mediated JAK/STAT signaling (Platanias 2005). The promoter regions of ISGs contain the Interferon-Stimulated Response Element (ISRE) through which type-1 IFNs control gene expression (Williams 1991). The NF-κB signaling in myeloid cells stimulates the expression of many cytokines and chemokines which play a crucial role in both acute and chronic inflammatory conditions (Liu et al. 2017). cGAS-STING signaling is also associated with many non-canonical downstream functions which are independent from the canonical pathways. For example, it is known that cGAS-STING signaling stimulates the function of the NLRP3 inflammasomes (Wang et al. 2020; Chen KQ et al. 2025) which are alarming PRRs producing mature IL-1β and IL-18 cytokines and an active gasdermin protein (Xu and Nunez 2023; Choi et al. 2025). It is evident that NLRP3 inflammasomes are important players in innate immunity responses in diverse pathological conditions. For instance, there are reports indicating that cytosolic escape of mtDNA activated NLRP3 inflammasomes through cGAS-STING signaling in mouse sepsis-mediated kidney injuries and macrophage-induced lung injuries (Luo et al. 2024; Peng et al. 2024). Moreover, it is known that the non-canonical cGAS-STING signaling pathway activated PERK signaling which enhanced cellular senescence and tissue fibrosis in mice (Zhang et al. 2022) (Fig. 1).

### AIM2 inflammasomes

Absent in melanoma 2 (AIM2) is a DNA sensor capable of forming an inflammasome complex (Hornung et al. 2009). It represents a vertebrate-restricted sensor with adaptive evolutionary changes occurring mainly in mammalian and primate lineages, in contrast to cGAS, whose homologs trace back to early eukaryotes and reflect a more ancient and widespread evolutionary origin (Cagliani et al. 2014; Wu et al. 2014). AIM2 is a member of the Pyrin and HIN domain-containing (PYHIN, also known as HIN200) family of proteins, which in humans also include PYHIN 1 (also known as IFIX), IFI16, and myeloid cell nuclear differentiation antigen (MNDA) (Hornung et al. 2009).

The PYHIN family of proteins comprises mainly cytosolic and nuclear DNA sensors, among which AIM2 uniquely couples dsDNA recognition directly to inflammasome activation, resulting in rapid maturation of pro-inflammatory cytokines and inflammatory cell death, distinct from interferon-inducing DNA-sensing pathways such as cGAS-STING (Hornung et al. 2009; Hopfner and Hornung 2020). AIM2 responds broadly to any cytosolic dsDNA including mtDNA (Xu et al. 2021; Boengiu et al. 2025), whereas cGAS activation depends strongly on DNA conformation and the ability of dsDNA to promote cGAS dimerisation rather than on its mere presence (Xu et al. 2024).

AIM2 directly binds dsDNA through its C-terminal HIN domain via electrostatic interactions between its positively chaged HIN domain and the negatively charged dsDNA (Hornung et al. 2009; Jin et al. 2012). Upon DNA binding, the N-terminal pyrin domain (PYD) of AIM2 interacts with the PYD of the adaptor protein ASC (apoptosis-associated speck-like protein containing a caspase recruitment domain, CARD) (Hornung et al. 2009; Jin et al. 2012). Subsequent recruitment of pro-caspase-1 via CARD-CARD interactions leads to the autoproteolytic activation of caspase-1 (Hornung et al. 2009; Shi et al. 2015). Active caspase-1 then matures IL-1β, IL-18 and gasdermins (Shi et al. 2015) (Fig. 1). In addition, the AIM2-ASC complex can promote NF-κB activation, a function preferentially observed for AIM2 in comparison to other PYHIN family members that do not form inflammasomes (Hornung et al. 2009). Moreover, AIM2 inflammasome activation primarily promotes pyroptosis, but alongside other innate immune sensors such as ZBP1, Pyrin, or NLR family CARD domain-containing protein 4 (NLRC4), it may also contribute to PANoptosis when inflammatory signaling is amplified, for example during infections, toxin exposure, or cytokines, such as TNF-α and IFN-γ (Lee et al. 2021; Oh et al. 2023; Xie et al. 2025) (Fig. 1). In addition to caspase-1, PANoptosis engages apoptotic and necroptotic components, including caspase-8 and RIPK3-MLKL signaling (Lee et al. 2021).

### IFI16 sensors

The IFI16 protein is an IFN-γ-inducible protein 16 which defends cells against infections through the recognition of foreign DNA (Cridland et al. 2012). While the IFI16 protein is ubiquitously expressed with a low-specificity in the majority of non-immune cells, its expression level is clearly higher in endothelial cells, macrophages, and leukocytes (Human Protein Atlas). Although the IFI16 factor is a potent sensor for foreign DNA, several investigators have revealed that it can also recognize cytosolic mtDNA (Matsui et al. 2021; Jahun et al. 2023; Deng et al. 2024). IFI16 is a multifunctional protein which has diverse functions in both the cytoplasm and the nuclei (Unterholzner et al. 2010; Jakobsen and Paludan 2014). The IFI16 protein is one of the PYHIN proteins containing the PYD protein–protein binding domain and two DNA-binding HIN domains. The IFI16 factor is normally located in the nuclei but after its acetylation it can be translocated into the cytoplasm (Li et al. 2012). The IFI16 sensor recognizes cytosolic dsDNA in a sequence-independent manner and subsequently it triggers proinflammatory responses. It is known that the activation of IFI16 can (i) trigger STING signaling and produce IFN-β and other cytokines (Almine et al. 2017), (ii) activate the AIM2 inflammasome and generate mature IL-1β, IL-18, and gasdermin factors (Jakobsen and Paludan 2014), and (iii) stimulate the expression of RIG-1 sensor (Jiang et al. 2021) (Fig. 1). There is abundant evidence that the IFI16 factor can collaborate with the cGAS-STING and RIG-1-mediated pathways to induce proinflammatory responses. For instance, Almine et al. (2017) demonstrated in human keratinocytes that the IFI16 factor interacted with the cGAS protein to promote cytosolic dsDNA-induced STING and IRF3 signaling. However, the interaction can also be suppressive, e.g., Zheng et al. (2020) reported that porcine IFI16 inhibited cGAS signaling by competing with dsDNA binding and thus preventing STING signaling. Moreover, there are studies indicating that the IFI16 factor can also cooperate with the RIG-1 pathway and stimulate the RIG-1-mediated type-1 IFN production in a STING-independent manner (Jiang et al. 2021; Shi et al. 2024). Although there does seem to be collaboration between IFI16 and cGAS-STING signaling, it is important to note that during an HSV infection, they generated crucially different responses (Diner et al. 2016).

In addition to inflammatory responses, the IFI16 factor controls a number of other functions associated with the maintenance of cellular homeostasis in response to diverse insults (Jakobsen and Paludan 2014). For instance, IFI16 can regulate the chromatin landscape, gene expression, DNA repair, PANoptosis, virus latency, and myeloid differentiation. Through the generation of type-1 IFNs, IFI16 not only controls the expression of immune genes but also regulates the functions of many non-immune ISGs in attempts to maintain tissue homeostasis. For example, Hotter et al. (2019) demonstrated that IFI16 suppressed the expression of HIV-1 genes by inhibiting the function of the Sp1 transcription factor and thus it was able to repress the reactivation of latent HIV-1. They also reported that IFI16 restricted the retrotransposition of LINE-1 elements. IFI16 can also suppress the replication of human cytomegalovirus (hCMV) by inhibiting the Sp1-mediated transactivation (Gariano et al. 2012). Moreover, it is known that IFI16 is a negative regulator of the transcription of human *p53*, *p21* and telomerase reverse transcriptase (*hTERT*) genes (Kwak et al. 2003; Song et al. 2010). In addition to the Sp1 transcription factor, the IFI16 factor can also interact with SUV39H1, an H3K9 methyltransferase, and many co-repressors, such as NuRD and Sin3A factors, and thus inhibit the expression of viral genomes (Roy et al. 2019; Ghosh et al. 2025). In human breast cancer cells, IFI16 has been demonstrated to prevent the repair of DNA double-stranded breaks by inhibiting the recruitment of repair factors (Ka et al. 2021). The IFI16 factor also has an intimate relationship with the PANoptosome cell death pathway in many heart diseases (Chang et al. 2024). These examples indicate that while the IFI16 factor does defend against viral infections, it can inflict harmful responses with respect to the maintenance of cellular homeostasis.

### ZBP1 sensors

The ZBP1protein is a sensor for dsDNA and RNA helices which are in a left-handed Z-conformation, i.e., opposite to the normal right-handed helical B structure (Wang and Vasquez 2007; Sahayasheela et al. 2025). The formation of Z-DNA is an active element in both nuclear and mitochondrial genomes appearing when there are disturbances in the integrity of the genome (Wang and Vasquez 2007). Mitochondrial stresses, such as oxidative stress, UV exposure, and doxorubicin treatment, promote the transition of mtDNA from its B-form to the Z-conformation and consequently activate ZBP1 signaling (Szczesny et al. 2018; Lei et al. 2023; Lai et al. 2024). Lei et al. (2023) demonstrated that the ZBP1 protein stabilized the Z-mtDNA released from the cardiac mitochondria of doxorubicin-treated mice and subsequently the ZBP1/Z-mtDNA structure assembled a complex with the cGAS protein. Consequently, this complex recruited the receptor-interacting serine/threonine-protein kinases 1 and 3 (RIPK1/3) which stimulated STAT1 signaling and augmented the production of type-1 IFNs (Lei et al. 2023). Similarly, it is known that both viral Z-RNA and host cell Z-RNA can also activate the ZBP1 protein, assemble the ZBP1/RIPK3 complex, and subsequently induce cell death (DeAntoneo et al. 2023; Yang et al. 2023; Yin et al. 2025). The ZBP1/RIPK3 complex is a signaling hub since it can trigger PANoptotic cell death, i.e., RIPK3 activates NLRP3 and subsequently (i) can induce GSDMD-mediated pyroptosis and (ii) trigger caspase 3/7-stimulated apoptosis. In addition, RIPK3 can also activate the mixed lineage kinase domain-like protein (MLKL) and induce necroptosis (Kuriakose and Kanneganti 2018; Zheng and Kanneganti 2020; Bi et al. 2025) (Fig. 1). Given that the expression of the ZBP1 protein is strongly induced by type-1 IFNs via IRF1 signaling (Kuriakose et al. 2018; Sharma et al. 2023), type-1 IFNs have a crucial role in the PANoptotic form of cell death not only in viral infected cells but also in many pathological conditions associated with an accumulation of cytoplasmic Z-DNA or Z-RNA (Kuriakose and Kanneganti 2018; Hao et al. 2022b; Maelfait and Rehwinkel 2023; Yang et al. 2023; Bi et al. 2025). Moreover, there are cytosolic inhibitors for ZBP1 signaling, such as nuclear/cytoplasmic adenosine deaminase acting on RNA 1 (ADAR1) and some viral proteins which can prevent ZBP1-mediated PANoptosis (Steain et al. 2020; Karki et al. 2021). Although the ZBP1 sensor has beneficial effects in the elimination of infected and tumor cells, its excessive activation and its ability to trigger PANoptotic cell death can lead to the appearance of harmful inflammatory responses in host tissues.

### RIG-1/MDA5 sensors

Cellular senescence is not only associated with a leakage of mtDNA but the release of mitochondrial dsRNA is also significantly increased from mitochondria in both immune and non-immune cells (Lopez-Polo et al. 2024; Victorelli et al. 2025). The bidirectional transcription of the circular mitochondria genome generates dsRNAs which are potent activators of cytosolic dsRNA sensors. Retinoic acid-inducible gene 1 (RIG-1, encoded by the *DDX58* gene) and melanoma differentiation-associated protein 5 (MDA5, encoded by the *IFIH1* gene) are the major cytosolic sensors for detecting viral and mitochondrial dsRNA (Rehwinkel and Gack 2020; Onomoto et al. 2021; Yoneyama et al. 2024). RIG-1 is an ATP-dependent DexD/H box RNA helicase which contains two CARD domains and the C-terminal RNA-binding domain. Through its CARD domains, the RIG-1 protein interacts with the mitochondrial antiviral signaling protein (MAVS, also referred as IPS-1) which is localized in mitochondrion-associated membranes (MAM). The MDA5 protein is a RIG-1-like dsRNA helicase that also interacts with the MAVS protein through the CARD domains. Activation of MAVS evokes the downstream stimulation of either (i) the TBK1/IKK-ε-driven production of type-1 IFN by activating IRF3/IRF7 transcription factors or (ii) the IKK-α/IKK-β-induced NF-κB signaling which triggers the secretion of inflammatory cytokines and chemokines (Rehwinkel and Gack 2020; Thoresen et al. 2021) (Fig. 1). The posttranslational regulation of the RIG-1/MDA5/MAVS proteins, e.g., by ubiquitination, acetylation, or phosphorylation, is an important mechanism controlling the function of these pathways (Chiang and Gack 2017; Quicke et al. 2017; Rehwinkel and Gack 2020). Although the RIG-1 and MDA5 sensors both recognize cytosolic mitochondrial dsRNA and stimulate the MAVS-driven pathways, there are differences in their expression patterns, target specificities, pathogen recognition, and connections to other signaling pathways (Loo et al. 2008; Brisse and Ly 2019). Indeed, it is known that dsRNA concurrently activates immune responses in cytoplasm both via the RIG-1 and MDA5 sensors (Lopez-Polo et al. 2024; Chen J et al. 2025; Victorelli et al. 2025).

The RIG-1 and MDA signaling pathways stimulate the expression of type-1 IFNs and proinflammatory cytokines in a similar manner as the cGAS-STING and ZBP1 pathways. Currently, it is known that in addition to its contribution in ensuring viral immunity, RIG-1 signaling regulates many non-immune events, e.g., it can control pyroptosis, apoptosis, and necroptosis, i.e., PANoptosis (Qiao et al. 2025). Several cytosolic mtDNA and dsRNA sensors possess a similar ability to trigger the PANoptotic cell death (Fig. 1). The RIG-1/MDA5/MAVS signaling pathways are stringently regulated because they can act as guardians of cell death or survival. Since the RIG-1 protein contains two CARD domains, it can activate caspase-1 which proteolytically cleaves the GSDMD protein and subsequently its different fragments can form membrane pores into mitochondria and the plasma membrane, thus evoking pyroptotic cell death (Jiao et al. 2025; Qiao et al. 2025). On the other hand, overexpression of RIG-1 can stimulate autophagy which in many contexts can act as a survival mechanism against viral infections (Lee et al. 2018). Lee et al. (2018) demonstrated that activation of RIG-1 signaling stimulated autophagy in mouse fibroblasts through the MAVS/TRAF6/Beclin-1 pathway. Another way to prevent excessive immune activation after viral infections is to transport the RIG-1/MDA5 factors for autophagic degradation via the selective autophagy pathway (Du et al. 2018; Hou et al. 2021). Nonetheless, an excessive antiviral ISG response can inhibit the activity of RIG-1 signaling, e.g., by expressing the IFN-α inducible IFI6 and IFI27 proteins which inhibit the activity of RIG-1 signaling (Villamayor et al. 2023a,b). Finally, there is a close crosstalk between the RIG-1 and cGAS-STING signaling pathways. For instance, interactions between the RIG-1/MAVS proteins and the STING platform can activate STING-mediated proinflammatory signaling (Zevini et al. 2017).

## Cytosolic DNA/RNA sensors as drivers of inflammation and the aging process

For decades, it has been speculated that mitochondria have a crucial role in the aging process. Initially, it was speculated that the production of ROS and mutations in mtDNA would be the main events in age-related degenerative processes. More recently, several breakthrough investigations have been published indicating that the sensors of viral immune defence can respond to a leakage of mtDNA and dsRNA and trigger type-1 IFN-driven inflammatory responses which induce cellular senescence and promote the aging process. Next, we will examine in detail the mechanisms through which different cytosolic DNA and RNA sensors can promote cellular senescence and the aging process.

### cGAS-STING signaling

There is convincing evidence that an accumulation of cytosolic dsDNA can stimulate cellular senescence via cGAS-STING signaling both in cultured cells and in aging tissues (Glück et al. 2017; Lan et al. 2019; Hu et al. 2022; Gulen et al. 2023; Salminen et al. 2025a, b). In particular, several studies have revealed that the leakage of mtDNA is a potent inducer of cellular senescence and it can trigger inflammatory responses via activation of cGAS-STING signaling (Aarreberg et al. 2019; Zhong et al. 2022; Hu et al. 2022; Gulen et al. 2023; Victorelli et al. 2023; Li et al. 2024) (Fig. 1). In fact, mtDNA-induced cGAS-STING signaling can stimulate the proinflammatory senescence-associated secretory phenotype (SASP), a hallmark of senescent cells in aged tissues (Yousefzadeh et al. 2020). Interestingly, there are also studies indicating that the proinflammatory cytokines, such as IL-1β and TNFα, can trigger the release of mtDNA and stimulate cGAS-STING signaling in human epithelial and myeloid cells (Aarreberg et al. 2019; Willemsen et al. 2021). Thus, it seems that in senescent cells, the expression and secretion of SASP factors can promote an mtDNA-induced cGAS-STING signaling to maintain an inflammatory state. Recently, Wu et al. (2024) demonstrated that cGAS-STING signaling could trigger the senescence state by stimulating the IRF3-induced activation of the p16INK4a-retinoblastoma (RB) pathway, a well-known inducer of cellular senescence. The crucial role of mtDNA-induced cGAS-STING signaling as an inducer of cellular senescence and a source of inflammatory responses has been confirmed with different research approaches. Moreover, there are investigations indicating that the inhibition of mtDNA-induced cGAS-STING signaling can ameliorate cellular senescence and downregulate the hallmarks of the SASP state (Hu et al. 2022; Zhong et al. 2022; Li et al. 2024; Zheng et al. 2024).

The accumulation of senescent cells in the aging process is associated with a clear decline in the activity of autophagy as well as mitophagy, a selective form of autophagy which removes aged and dysfunctional mitochondria (Cuervo et al. 2008; Salminen and Kaarniranta 2009; Bakula and Scheibye-Knudsen 2020; Chen et al. 2020). As described above, an age-related decline in the integrity of mitochondria increases the release of mtDNA across different membrane pores and subsequently it activates alarming immune responses via cGAS-STING signaling. There are a number of experimental studies indicating that an inhibition of mitophagy can evoke a leakage of mtDNA which consequently stimulates cGAS-STING signaling, whereas an increase in mitophagic activity inhibits mtDNA release and prevents the activation of cGAS-STING signaling thus attenuating the appearance of inflammatory responses (Willemsen et al. 2021; Hu et al. 2022; Zhong et al. 2022; Jimenez-Loygorri et al. 2024; Li et al. 2024; Zhou et al. 2025). For instance, Zhong et al. (2022) demonstrated that macrophages from aged mice displayed an increased cytosolic leakage of mtDNA which promoted inflammatory responses via an activation of cGAS-STING signaling. They reported that the age-related decline in PINK1/Parkin signaling attenuated mitophagy and thus promoted mtDNA-mediated cGAS-STING signaling. Accordingly, Gulen et al. (2023) revealed that in aged murine microglia an increased level of cytosolic mtDNA activated cGAS-STING signaling and promoted neuroinflammation and degeneration in mouse brain. Moreover, there is clear evidence emerging from progeroid pathology indicating that defects in mitophagy and an increase in cGAS-STING signaling accelerate the aging process in Hutchinson-Gilford syndrome and Zmpste24 mouse model (Li et al. 2024; Sun et al. 2024). It seems that an age-related loss of mitochondrial integrity triggers a leakage of mtDNA that promotes inflammaging via cGAS-STING signaling.

### AIM2 inflammasomes

In aging cells, mitochondrial dysfunction promotes the accumulation of mtDNA in the cytoplasm thereby increasing cytosolic DNA availability that can engage AIM2 and reinforce inflammaging-associated features including the SASP state in aged tissues (Victorelli et al. 2023; Boengiu et al. 2025). mtDNA has been shown to promote AIM2 inflammasome activation in sterile inflammatory conditions, providing a direct mechanistic link between age-associated mtDNA and AIM2 engagement (Bae et al. 2019). Functionally, AIM2 is classically linked to rapid inflammasome assembly and cytokine maturation (IL-1β and IL-18), which can sustain chronic inflammation in tissues, whereas cGAS-STING and IFI16 pathways are commonly associated with interferon signaling and transcriptional programs linked to cellular senescence, although IFI16 also contributes to inflammasome activation and interferon-independent functions (Song et al. 2008; Almine et al. 2017; Glück et al. 2017). Accordingly, under mitochondrial dysfunction, AIM2 inflammasome signalling may be biased towards sustaining age-related sterile inflammation rather than driving acute host-defence responses (Bae et al. 2019; Christgen and Kanneganti 2020). In this way, AIM2 provides a plausible mechanistic route by which age-associated mitochondrial damage is translated into persistent, tissue-burdening inflammation. In line with this complexity, cytosolic dsDNA recognition is not uniform across tissues. For example, cGAS-STING signaling, but not AIM2 inflammasome activation, contributed to nucleus pulposus (NP) cell senescence, a process implicated in intervertebral disc degeneration and thereby in age-associated low back pain (Zhang 2024c).

In addition to chronic ligand exposure, other age-related changes such as an overall pro-inflammatory environment, e.g., due to increased basal NF-κB activation, may support increased inflammasome signaling during aging (Franceschi et al. 2018). However, AIM2 inflammasome activation is highly cell-type and context-dependent, and age-related changes appear heterogeneous rather than uniformly increased. In many cases, AIM2 inflammasome activation has been reported to increase, such as in human prostate epithelial cells upon cellular senescence, premature immunosenescence in psoriasis patients, age-related cognitive dysfunction in mice, and in skin aging promoted by nano-sized microplastics (Sahmatova et al. 2017; Panchanathan et al. 2021; Ye et al. 2023; Han et al. 2024). In addition, aging increases mutations in clonal hematopoiesis, and AIM2 has been linked through JAK2^V617F^ and ASXL1 mutations to atherosclerosis (Yalcinkaya and Tall 2024). In contrast, AIM2 expression and activity were shown to decrease in monocytes during healthy aging, which may be associated with increased infection sensitivity (Wang et al. 2016). Low AIM2 inflammasome activity, however, cannot be considered equivalent to healthy aging. The expression of AIM2 was observed to increase during aging in healthy gingival mucosa, and it was hypothesized that the AIM2 inflammasome could maintain a healthy state in the periodontium (Ebersole et al. 2016). However, in a later study, Hao et al. (2026) showed that AIM2-mediated senescence in human gingival fibroblasts exacerbate inflammaging in periodontitis. Together, these findings are consistent with a dual role in which baseline AIM2 activity may contribute to tissue homeostasis, whereas sustained activation in disease contexts can promote inflammaging. In line with this, the levels of DNA sensors appear to be very delicately regulated during aging. In a study with normal human fibroblasts, the levels of IFI16 were increased in old but reduced in senescent cells, whereas AIM2 levels did not increase until the cells reached senescence (Duan et al. 2011). Collectively, the literature suggests that AIM2 is not a universal marker of”healthy” or”pathological” aging but a regulated node whose effect depends on the cell type, the source and persistence of cytosolic DNA, and the inflammatory milieu.

In age-related diseases, AIM2 signaling may exert mixed and model-dependent effects. AIM2 has been epigenetically and transcriptionally associated with Alzheimer’s disease (Nazarian et al. 2020). In an Alzheimer mouse model, AIM2 knock-out mitigated β-amyloid deposition and microglial activation but did not improve spatial memory and increased inflammatory cytokine expression (Wu et al. 2017). In post-mortem human brains from patients with intermediate Alzheimer’s disease, AIM2 expression was increased and the receptor was clustered in neuritic plaques and co-localized with dense ASC expression (de Rivero Vaccari et al. 2026). Clarifying the role of AIM2 in aging will likely require cell-type-resolved, longitudinal analyses that distinguish basal homeostatic signaling from sustained inflammasome activation driven by chronic cytosolic DNA exposure.

### IFI16 signaling

There is abundant evidence that the expression of the IFI16 factor is significantly upregulated in senescent cells (Xin et al. 2003, 2004; Song et al. 2008; Kim et al. 2009). In fact, there are reports indicating that IFI16 might even be an inducer of cellular senescence. For example, Song et al. (2008) demonstrated that the expression of p53 significantly upregulated the expression of the IFI16 protein in human primary fibroblasts and human Saos2 cells. They revealed that the p53 factor bound to the promoter region of the *IFI16* gene which contains a cognate binding site for p53. They also reported that doxorubicin treatment robustly induced the binding of p53 and strongly stimulated the transcription of the *IFI16* gene. Accordingly, Xin et al. (2004) observed that the expression of IFI16 was clearly upregulated in human senescent fibroblasts when they were compared to young cells. Interestingly, they revealed that a reduction in the expression of IFI16 was associated with an immortalization of fibroblasts. Moreover, a knockdown of IFI16 expression decreased the expression levels of SA-β-gal and p21WAF1, markers of cellular senescence, and extended the replicative lifespan of human fibroblasts. In addition, Clarke et al. (2010) demonstrated that an overexpression of IFI16 in human MCF-7 cells decreased cellular proliferation and induced a senescence-like morphology displaying an increased level of the SA-β-gal protein. They also reported that the IFI16 protein and the human telomerase reverse transcriptase (hTERT) physically interacted both in vitro and in vivo and this interaction inhibited the activity of telomerase in senescent MCF-7 cells. Song et al. (2010) also confirmed that an increased level of IFI16 protein in human WI-38 fibroblasts robustly downregulated the expression of the *hTERT* gene and reduced cell proliferation. These studies clearly indicate that the IFI16 factor has a crucial role in the regulation of cellular senescence.

Given that the *IFI16* gene is an IFN-γ-inducible gene, age-related changes in the production of IFN-γ might affect the responses of IFI16 signaling with aging. However, it seems that the aging process is associated with complex changes in the production of IFN-γ, e.g., aging evokes a clear decline in its production of IFN-γ by mononuclear cells of human blood thus increasing the individual’s sensitivity to infections (Candore et al. 1993; Ouyang et al. 2002; Feng et al. 2021). Nonetheless, within tissues aging induces a clonal expansion of T cells, macrophages, and neutrophils, which display a robust production of IFN-γ (Singh et al. 2011; Kim et al. 2013; Dulken et al. 2019; Ashour et al. 2023). These observations indicate that IFN-γ is an important player in the age-related tissue degeneration although IFI16 is only one of the IFN-γ-responsive genes. However, as described above, IFI16 is a potent enhancer of cellular senescence and thus an age-related increase in IFN-γ production by certain immune cells might expand cellular senescence within aged tissues. IFI16 can promote the aging process via different mechanisms, e.g., IFI16 can (i) inhibit the function of the Sp1 factor and thus affect epigenetic regulation (Hotter et al. 2019), (ii) stimulate inflammatory responses via activation of both STING and NLRP3 inflammasomes (Jakobsen and Paludan 2014), (iii) promote the attrition of telomeres by inhibiting the activity of hTERT (Clarke et al. 2010), (iv) augment extracellular remodelling and enhance fibrosis (He et al. 2025), and (v) increase cellular senescence and PANoptotic cell death (Xin et al. 2004; Song et al. 2008; Chang et al. 2024) (Fig. 1). Choubey and Panchanathan (2016) and Choubey (2022) have reviewed in detail the many properties of IFI16 in the regulation of the aging process.

### ZBP1 signaling

There are several reports indicating that the generation of Z-DNA and Z-RNA is associated with cellular senescence in different experimental models (Yang et al. 2023; Kong et al. 2025; Wang et al. 2025; Qiao et al. 2026). For instance, Qiao et al. (2026) demonstrated that the treatment of human vascular endothelial cells with tetrachlorobisphenol A (TCBPA) increased the level of mitochondrial ROS and promoted the generation of Z-mtDNA in mitochondria. Subsequently, they observed a concentration-dependent increase in the expression of the ZBP1 protein and also the activation of RIPK3 signaling, leading to cellular senescence. The activation of ZBP1/RIPK3 signaling in endothelial cells was associated with an increase in the expression of many pro-inflammatory cytokines as well as p16, p21, p53, and γ-H2AX, common markers of cellular senescence. Treatment of endothelial cells with an inhibitor of mitochondrial ROS generation clearly prevented the TCBPA-induced cellular senescence. Accordingly, Szczesny et al. (2018) revealed that a mild mitochondrial oxidative stress induced damage to mtDNA in pulmonary epithelial cells and simultaneously increasing production of inflammatory cytokines. They reported that a leakage of damaged mtDNA induced the activation of ZBP1 and stimulated signaling through the ZBP1/TBK1/IRF3 pathway. Interestingly, they reported that damaged mtDNA was released from senescent cells via membrane-bound extracellular vesicles (exosomes) which induced paracrine inflammatory alterations both in untreated epithelial cells and in vivo in mice. This is in accordance with observations indicating that senescent cells secrete extracellular vesicles and in this way they can expand the zone of cellular senescence via a bystander effect (Nelson et al. 2012; da Silva et al. 2019; Salminen et al. 2020).

The ZBP1 protein is a multipotent factor since it is linked with several signaling pathways driving not only many proinflammatory responses via type-1 IFN and NLRP3 signaling but it can also impair DNA repair processes (Shi et al. 2026) and induce PANoptotic cell death which is associated with several age-related diseases, such as cardiovascular diseases and many neurodegenerative diseases (Xiang et al. 2024; Han et al. 2025; Hou et al. 2025) (Fig. 1). Recently, Radak and Fallahi (2023) reviewed in detail the role of the ZBP1 factor in a variety of age-related cellular dysfunctions. This raises an interesting question what is the role of the ADAR1 protein, an inhibitor of the ZBP1 factor (Karki et al. 2021), in the regulation of cellular senescence and the aging process. Hao et al. (2022a) demonstrated that the expression of the ADAR1 protein was robustly downregulated in senescent human IMR90 fibroblasts. They also reported that the expression level of the ADAR1 protein was strongly reduced in many tissues of aged mice, such as brain cortex, ovary, and intestine. Hao et al. (2022a) revealed that ADAR1 controlled cellular senescence via SIRT1/p16INK4a signaling. The functions of the ZBP1 or RIG-1 sensors were not examined and thus their involvement needs to be clarified.

### RIG-1/MDA5 signaling

The leakage of mitochondrial dsRNA is a potent inducer of cellular senescence (Lopez-Polo et al. 2024; Victorelli et al. 2025). Victorelli et al. (2025) demonstrated that the cytosolic fraction of senescent human fibroblasts displayed a significant increase in mitochondria-derived dsRNA as well as a robust upregulation in the expression of RIG-1 and MDA5 factors at both the mRNA and protein levels. They demonstrated that a transfection of proliferating human fibroblasts with mitochondrial dsRNA significantly increased the expression of the RIG-1 and MDA5 proteins concomitantly with common markers of SASP. Victorelli et al. (2025) confirmed that in human fibroblasts an increase in RIG-1 and MDA5 signaling promoted the generation of the proinflammatory SASP state, i.e., mitochondrial dsRNA in the cytoplasm is a driver of the proinflammatory SASP state (Fig. 1). The activation of MAVS signaling was also associated with the development of the senescent state. They also reported that the aging process robustly increased the expression levels of RIG-1 and MDA6 in mouse heart, kidney, liver, and spleen concurrently with p16 and p21, common markers of the SASP state. Moreover, Lopez-Polo et al. (2024) demonstrated that a leakage of mitochondrial dsRNA into the cytoplasm was an important driver of the SASP state in several human and mouse cell lines in many experimental senescence models. They emphasized that the activation of MAVS was a key inducer of the proinflammatory state of senescent cells. Lopez-Polo et al. (2024) also reported that the age-related expression levels of the *RIG-1*, *MDA5*, and *MAVS* genes as well as that of the *STING* gene were significantly upregulated in human brain and liver. These studies clearly indicated that a leakage of mitochondrial nucleic acids, both mtDNA and dsRNA, has profound significance not only in cellular senescence but also in the aging process.

There is clear evidence that a leakage of mitochondrial dsRNA and the subsequent activation of RIG-1 and MDA5 sensors are associated with many inflammatory and age-related diseases (Wang et al. 2022; Chen et al. 2025a, b). Disturbances in mitochondrial dynamics and oxidative stress can trigger a leakage of mitochondrial dsRNA from mitochondria and induce both acute and chronic inflammatory responses (Yasukawa et al. 2009; Lopez-Polo et al. 2024; Xu et al. 2025). Yasukawa et al. (2009) reported that in human HEK293 cells, mitofusin 2 (MFN2), an enhancer of mitochondrial fusion, interacted with the MAVS protein and inhibited the function of the RIG-1/MDA5 pathway, whereas the loss of the MFN2 protein enhanced RIG-1 and MDA5 signaling. In rat skeletal muscle, the expression of MFN2 declines with aging (Sebastian et al. 2016) which might enhance the activity of RIG-1 and MDA5 signaling and lead to the development of the SASP state. Interestingly, Lopez-Polo et al. (2024) demonstrated that the expression levels of MFN1 and MFN2 were elevated in human senescent fibroblasts. The silencing of MFN1 suppressed the SASP state, whereas the inhibition of MFN2 significantly increased the expression of the SASP factors. These studies indicated that the mitofusins possess antagonistic properties in the regulation of the SASP state. In addition, it is known that MAVS can control mitochondrial morphology, whereas mitochondrial dynamics can regulate MAVS/STING-mediated signaling (Castanier et al. 2010). Currently, the role of the crosstalk between RIG-1/MDA5/MAVS signaling and mitochondrial dynamics in the regulation of cellular senescence and the aging process needs to be clarified. Liu et al. (2011) demonstrated that RIG-1 signaling induced the SASP state in human fibroblasts and endothelial cells. Interestingly, they demonstrated that the Klotho protein, a well-known anti-aging protein, suppressed the expression of proinflammatory mediators by interacting with the RIG-1 protein and thus inhibiting MAVS-mediated signaling. It is known that Klotho has diverse functions through which it is able to extend the healthspan and lifespan (Kuro-O 2019).

## During aging mitochondria turn into saboteurs inside eukaryotic cells

During aging, clear structural and functional alterations occur in mitochondrial population. Two important unresolved questions are whether these changes are inherent to the mitochondrial population or are they a byproduct of an aging microenvironment? Interestingly, it is known that bacteria also undergo cellular senescence and an aging process (Nyström 2007; Ksiazek 2010; Steiner 2021). However, the aging process of bacteria is commonly associated with an asymmetric division where cellular damages are segregated to one daughter cell, while the other daughter cell can be considered as a rejuvenated one. Mitochondria also divide in an asymmetrical manner but it is not known whether there exists a segregation of damaged structures between the progeny. Moreover, in many cell types mitochondria form a dynamic structural network through fusion and fission events which are driven by the cellular redox state (Singh et al. 2025).

There is now evidence that the mitochondria of aged/senescent cells are more prone to generate ROS as compared to those of young cells (Martini and Passos 2023). Consequently, an increase in ROS production enhances mitochondrial permeability which stimulates the release of ROS and mtDNA from mitochondria (Rottenberg and Hoek 2021; Zhao et al. 2021). Moreover, the rate of mitochondrial production of ROS is significantly lower in long-lived species (Gomez et al. 2023). Increased ROS generation has also been associated with stress-induced cellular senescence in plants (Jajic et al. 2015). For decades, it has been known that in mammalian cells the electron transfer chain (ETC) complexes I-III can generate ROS in response to diverse stresses (Chen et al. 2003; Zhao et al. 2019). There is also abundant evidence that the aging process decreases the efficiency of electron transport through the ETC and simultaneously increases the production of ROS (Kwong and Sohal 2000; Ferguson et al. 2005; Tatarkova et al. 2011). In addition, recent studies have revealed that with aging a reverse electron transfer (RET) can take place at complex I which increases ROS generation and promotes the conversion of NAD^+^ to NADH (Rimal et al. 2023; Bao et al. 2025). Currently, the ETC pathway is viewed as an important drug discovery target in attempts to find drugs able to prolong longevity (Bao et al. 2025; Radovic et al. 2025). Interestingly, studies on the role of ETC in bacterial senescence/aging have revealed that the aging process is associated with an increase in ROS generation, an aggregation of waste products, and a decline in energy metabolic pathways, i.e., similar events as occur in the aging process in mammalian cells (Ksiazek 2010; Steiner 2021). However, following endosymbiosis, there have appeared many modifications in the mitochondrial ETC pathway (Berry et al. 2003). Moreover, bacterial aging is an important research area because our gut microbiome contains trillions of bacteria which affect our health (Sender et al. 2016). There are several investigations indicating that age-related changes in the gut microbiome have a significant impact on the host immunity and it has been claimed that the microbiome can modulate the aging process (Bosco and Noti 2021; Ghosh et al. 2022).

Over three decades ago, Hayflick (1994) published the book “How and Why We Age”, profound questions which have mostly remained unresolved so far. However, many novel signaling mechanisms have been discovered during the last thirty years. For instance, the dsDNA sensors associated with inflammatory responses, especially type-1 IFN-related signaling, are interesting since they provide a connection between the defence against infections and the age-related leakage of mtDNA from mitochondria. There is robust evidence that the premature aging process is associated with many chronic infections, e.g., HIV and cytomegalovirus (Kusko et al. 2012; Cohen and Torres 2017; Higdon et al. 2021; Johnson et al. 2025). It is known that HIV and several other infections stimulate cGAS-STING signaling (Gao et al. 2013; Lio et al. 2016; Zhang et al. 2024b). This implies that the activation of dsDNA sensors can promote the aging process via the activation of cellular senescence and the development of chronic low-grade inflammation. It seems that the endosymbiosis model of aging might provide answers to both questions, i.e., “how and why we age” if we examine these subjects from an evolutionary perspective. Given that viruses evolved much earlier than the endosymbiotic event (Koonin et al. 2022), it is likely that dsDNA sensor mechanisms existed before the endosymbiosis between α-proteobacteria and archaeal cells, about 1.5–2 billion years ago. Endosymbiosis provided an aerobic energy metabolic powerhouse for eukaryotic cells but simultaneously it exposed cells to harmful inflammatory responses promoting the aging process. The question “why do we age” has sparked diverse evolutionary and more philosophic theories (Kirkwood 2002; Mc Auley 2024). However, it is likely that the evolution and development of organisms require that parent organisms will die to leave space for development of new progenies. In this sense, endosymbiosis provided a brilliant mitochondria-related mechanism inside host cells to drive the aging process and death of organisms.

## Conclusions

For decades, diverse theories on the role of mitochondria in the aging process have been proposed although there has been critical debate on different mechanisms. Mitochondria are the powerhouses of the organism but with aging, their properties become less efficient and disturbed. The endosymbiotic theory of aging emphasizes that mitochondria are involved in the aging process although it does not reveal any straightforward mechanism to account for this phenomenon. However, when viewed from an evolutionary perspective, one can appreciate why there is a need for an aging process in multicellular organisms. It is known that before the endosymbiotic event there existed a close coevolution between viruses and host archaeal cells. This means that host cells possessed mechanisms to defend themselves against viral DNA and RNA. It is likely that cytosolic nucleic acid sensors against viral attacks originated during this time period. The endosymbiosis changed the situation because host cells had to have a capability initially to manage approprietaly bacterial genes and subsequently cope with the mitochondrial genes. Mitochondria are very dynamic organelles and disturbances in their homeostasis, such as occur with aging, trigger a leakage of mtDNA/dsRNA into the cytoplasm where they activate multiple sensors stimulating the generation of type-1 IFNs and diverse cytokines. An increase in the secretion of type-1 IFNs is a typical immune response against viral attacks but their release also increases in cellular senescence and the aging process. For instance, Benayoun et al. (2019) demonstrated that the expression of genes stimulated by type-1 IFN-α, i.e., the IFN-stimulated genes (ISG), was most enriched in several tissues of old mice. Many other investigators have also revealed that type-1 IFNs are crucial enhancers of cellular senescence as well as the aging process and progeria (Frisch and MacFawn 2020; D’Souza et al. 2021; Cao 2022; Cancado de Faria 2023; Wang et al. 2024). This indicates that an age-related increase in type-1 IFN signaling could be attributed to a leakage of mtDNA/dsRNA from mitochondria. Endosymbiosis of mitochondria might also resolve the question on evolution of the aging process in multicellular organisms because with aging mitochondria seem to turn into saboteurs inside eukaryotic cells and thus they provide an endogenous source which drives the aging process.

## Data Availability

No datasets were generated or analysed during the current study.
